# Molecular Dynamics Simulations of Melting Iron Nanoparticles with/without Defects Using a Reaxff Reactive Force Field

**DOI:** 10.1038/s41598-020-60416-5

**Published:** 2020-02-25

**Authors:** Junlei Sun, Pingan Liu, Mengjun Wang, Junpeng Liu

**Affiliations:** 1 College of Aerospace and Civil Engineering, Harbin Engineering University, Heilongjiang Province, Harbin City, China; 2 Key Laboratory of Dual Dielectric Power Technology, Hebei Hanguang Industry Co. Ltd, Handan, 056017 China

**Keywords:** Materials science, Nanoscale materials, Nanoparticles

## Abstract

Molecular dynamics simulations are performed to study thermal properties of bulk iron material and Fe nanoparticles (FNP) by using a ReaxFF reactive force field. Thermodynamic and energy properties such as radial distribution function, Lindemann index and potential energy plots are adopted to study the melting behaviors of FNPs from 300 K to 2500 K. A step-heating method is introduced to obtain equilibrium melting points. Our results show ReaxFF force field is able to detect size effect in FNP melting no matter in energy or structure evolution aspect. Extra storage energy of FNPs caused by defects (0%-10%) is firstly studied in this paper: defects will not affect the melting point of FNPs directly but increase the system energy especially when temperature reaches the melting points.

## Introduction

Nanotechnology deals with the materials in nanometer size (10^−9^ m) which usually exhibit physical and chemical properties compared with their bulk or micro-size counterparts. Nanoparticles show peculiar optical, magnetic, electronic and thermo properties. Such performance should be attributed to their high surface/volume ratio and unique characteristic structure. Among all metal nanomaterials, Fe nanoparticles (FNP) stand out from others because its nontoxic, biodegradability and magnetic properties^1^. Recently, nanosized iron and its oxides have been extensively used in propulsion and energy-conversion applications due to their outstanding exothermic redox reaction characteristics, excellent high-temperature and corrosion properties. Although iron is not easily ignited at room temperature, combinations of iron and other nano metal or organic compounds have proved to be a promising energetic materials^2–4^. Thermodynamic properties of FNP will directly affect the energy release performance of synthesized nanomaterials. Additionally, the melting temperature of FNP is one of the the most basic and critical properties. However, due to the difficulty in determining the melting point from experiments, molecular dynamics simulations (MD) are becoming the main method to study thermal properties of nanoparticles^5^. Over the past 15 years, MD contributes to the melting of FNP in various ways.

Shubuta and Suzuki studied melting and nucleation of bcc FNPs by using Finnis-Sinclair potential. They introduce solid-liquid balance method to detect melting points of FNPs. Surface pre-melting and cooling nucleation process were observed in their work^6^. Joshi *et al*. studied melting processes of FNP and Ni particles by using EAM potentials. Their results prove size-dependent melting rule and there exists a linear relationship between melting temperature and 1/D (diameter of nanoparticles)^7^. Wen and Sun *et al*. focus on the structure evolution process of FNP form fcc to bcc during the heating process by using Finnis-Sinclair potential. Their results reveal fcc FNPs will undergo structural transformation and completely turn into bcc structure before temperature reaching the melting point^8^. Shu *et al*. firstly adopt replica-exchange molecular dynamics (REMD) to study FNP melting process which efficiently avoids superheating and undercooling phenomenon during simulations. Their simulation proves that melting points and particle size do not change linearly. Their results are in correspond with theory models and predict bulk iron melting points successfully according to experiments^5^. Wu and Shen *et al*. fcc by using Sutton-Chen potential. They detect magic atom number during the melting process: FNP will not obey traditional solid-liquid phase transition law when the number of cluster atoms is less than 113 and they attribute different structure evolution of fcc FNPs to the different cooling rates before heating^9^. Lavruk *et al*. studied the melting behavior of FNP which range from 3 to 8 nm by using EAM potential and energy change versus time was discussed in that paper^10^.

As introduced above, size dependence of FNP melting has been studied thoroughly, however, explanation of FNP melting in energy perspective and melting rule of FNPs with defects still need further discussion. Moreover, because of the difference of force field used and simulation methods, the melting points of FNPs obtained by researchers are also inconsistent. To the best of our knowledge, little is about using the ReaxFF force field which allows reactions occur to describe the melting process of FNP. In this paper, we perform canonical ensemble (NVT) MD simulations for FNP with perfect bcc lattice and different defect concentrations. The rest of this paper is compiled as follows: Section 2 introduces the force field used in this paper and MD settings. Section 3 is a results discussion section. The conclusion is arranged in section 4.

## Methods

### ReaxFF force field

All MD simulations in this paper are carried out by using the ReaxFF force field which is a method based on the principles of quantum mechanics (QM). The general expression for total energy in ReaxFF force field is defined as Eq. (1):

1 $${E}_{system}={E}_{bond}+{E}_{over}+{E}_{under}+{E}_{lp}+{E}_{val}+{E}_{tors}+{E}_{vdWaals}+{E}_{Coulomb}$$

where E_system_ represents the total system energy. The bond (E_bond_), overcoordination (E_over_), lonepair (E_lp_), valence angle (E_val_), van der Waals (E_vdWaals_), and Coulomb (E_Coulomb_) energy terms contribute to total energy in various degrees. Additionally, nonbonded interactions are calculated independently from other bonded interaction terms which means no contradictory data shared or transferred between them.

Unlike other traditional or empirical force fields which usually pre-define bonded information between atoms, ReaxFF introduces bond-order (BO) system to detect and calculate bonded interactions which directly affect the occurrence and process of interactions. Equation (2) displays the BO expressions:

2 $$B{O}_{ij}=B{O}_{ij}^{\sigma }+B{O}_{ij}^{\pi }+B{O}_{ij}^{\pi \pi }=\exp [{p}_{bo1}{(\frac{{r}_{ij}}{{r}_{0}^{\sigma }})}^{{p}_{bo2}}]+exp[{p}_{bo3}{(\frac{{r}_{ij}}{{r}_{0}^{\pi }})}^{{p}_{bo4}}]+exp[{p}_{bo5}{(\frac{{r}_{ij}}{{r}_{0}^{\pi \pi }})}^{{p}_{bo6}}]$$

where BO_ij_ is the bond order between atom i and j, r_0_ denotes equilibrium bond lengths and p_bo_ terms are empirical parameters. The equation is continuous and contains no discontinuities through transitions between σ, π and ππ bond characters. What’s more, bond order does not include any pair interactions. Instead, the force field ll build neighbor lists for every atom from the simulation beginning to avoid spurious bond characters. Other excessive close-range nonbonded interactions are also avoided by calling shielding terms. Bond order neighbor lists will be updated every iteration step to recalculate all bonded interactions.

After nearly two decades development, ReaxFF has been applied successfully in various computational chemistry field: heterogeneous catalysis, vanadium catalysts, atomic layer deposition and high energy reaction systems in nanoscale^11–14^.

### Simulation setup

The simulation box is set up as a 100 Å × 100 Å × 100 Å cubic box with periodic boundaries in three orthogonal directions. The equations of atomic motion are integrated by the Verlet-Velocity algorithm^15^. In this work, all MD simulations are performed under canonical ensemble (NVT). Berendsen heating-bath is adopted to modify system temperature and the Berendsen damp factor is 1.0 fs which means to relax system temperature every timestep. It has been reported that 0.5-1.0 fs is a reasonable parameter range in ReaxFF MD simulations because bond-order mechanism is able to detect reactions and update neighbor lists timely^16^. Considering only one substance exist in the simulation system and no violent reactions occur, timestep of 1.0 fs is enough to describe thermal motion behaviors of metal atoms.

The heating rate is another important factor which directly affects the thermodynamic results. Taking both computation cost and accuracy of recording motions of atoms into account, we test three heating rates on a 2 nm FNP: 0.001 K/fs, 0.01 K/fs and 0.1 K/fs. Our test results show that the first two rates could obviously show the melting point by monitoring the change of energy, however, a 0.1 K/fs heating rate will miss melting points due to its excessive heating rate. Therefore, the heating rate applied in this paper is on the order of 0.01 K/fs.

All FNPs are cut from bulk material with bcc lattice 2.8664 Å and placed in the center of the simulation box. The information of FNPs is listed in Table 1. The 3 nm FNP with fcc lattice is prepared to achieve a comparative study.

**Table 1:** Information of FNP models for melting simulations.

Diameter (nm)	2.0	2.5	3.0	3.5	4.0
Lattice	bcc	bcc	bcc	bcc	bcc
Atoms	338	785	1218	2073	2809

Additionally, 3 nm FNPs with defect concentration ranges from 0% to 20% are also prepared for the study of melting point and energy changes caused by defects and relevant information is listed in Table 2. To simplify the complexity of modeling, all defects are treated as point defects. The defects are generated by deleting a certain number of atoms randomly.

**Table 2:** Atom information of 3 nm bcc FNPs with different defect concentrations.

Defect concentration	0%	2%	4%	6%	8%	10%
Atom number	1218	1194	1169	1144	1120	1096

All cases share the same heating procedure which is performed as follows: firstly, the velocities of all atoms are set at 300 K through the Gaussian distribution and minimization process using steepest descent method is performed for each FNP to obtain the reasonable configurations. Then, a 50 ps relaxation process at 300 K before heating is performed to fully relax the structure. The annealed process is omitted because the main object of this study is bcc FNPs and it is necessary to maintain their bcc structure before melting. The systems are heated from 300 K to 2500 K to ensure the melting process occurs.

To eliminate the influence of high heating rates on FNP, we adopt step-heating strategy. Because the melting point of iron is much higher than that of aluminum, we set the heating interval as 25 K. A 100 ps equilibrium process is performed to record analytical data at the current temperature after the system reaching the target temperature.

All MD simulations are carried out by LAMMPS with USER-REAXC package^17,18^. OVITO and VMD are chosen as visualization and post-processing software respectively^19,20^.

## Results and Discussions

### Tests on bulk fe melting

Firstly, we test the force field on bulk material. Both bulk irons of bcc and fcc are built in a 45×45×45 Å simulation box for melting and lattice structure tests. The system is relaxed under 300 K for 20 ps. After that, the system is heated to 2500 K at a rate of 2.5×10^12^ K/s by using step-heating strategy. During the relaxation process under 300.0 K the lattice constant for both models are almost the same as the initial value which is shown in Table 3. Such results show the ReaxFF force field used in this paper can maintain reasonable structures for iron bulk material.

**Table 3:** The lattice constant before/after relaxation process.

Lattice constant (Å)	Initial setting	After 500 ps relaxation
bcc	2.8664	2.8574
fcc	3.6583	3.6852

**Figure 1:**
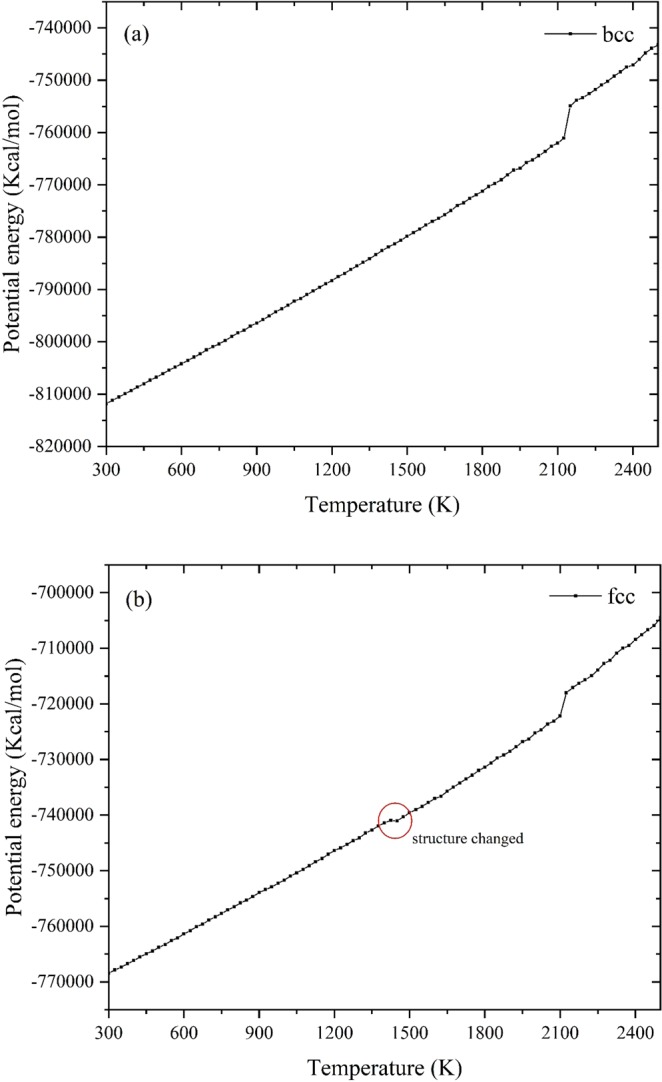
Potential energy curves for (**a**) bcc and (**b**) fcc bulk iron during the melting tests.

Heating rate 0.01 K/fs is used to simulate melting behaviors of bulk material from 300.0 K to 2500.0 K. The potential energy versus temperature curves are shown in Fig. 1.

It is worth noting that, for fcc structure in Fig. 1(b), the potential curve becomes flat suddenly at 1425 K which indicates the structure is changing from 1425 K to 1450 K. As all known, fcc structure is a metastable structure for bulk iron. Although fcc structure nanoparticles are not the focus of this paper, the ReaxFF still detects the energy fluctuations caused by structural changes which proves the applicability of the force field further.

**Figure 2:**
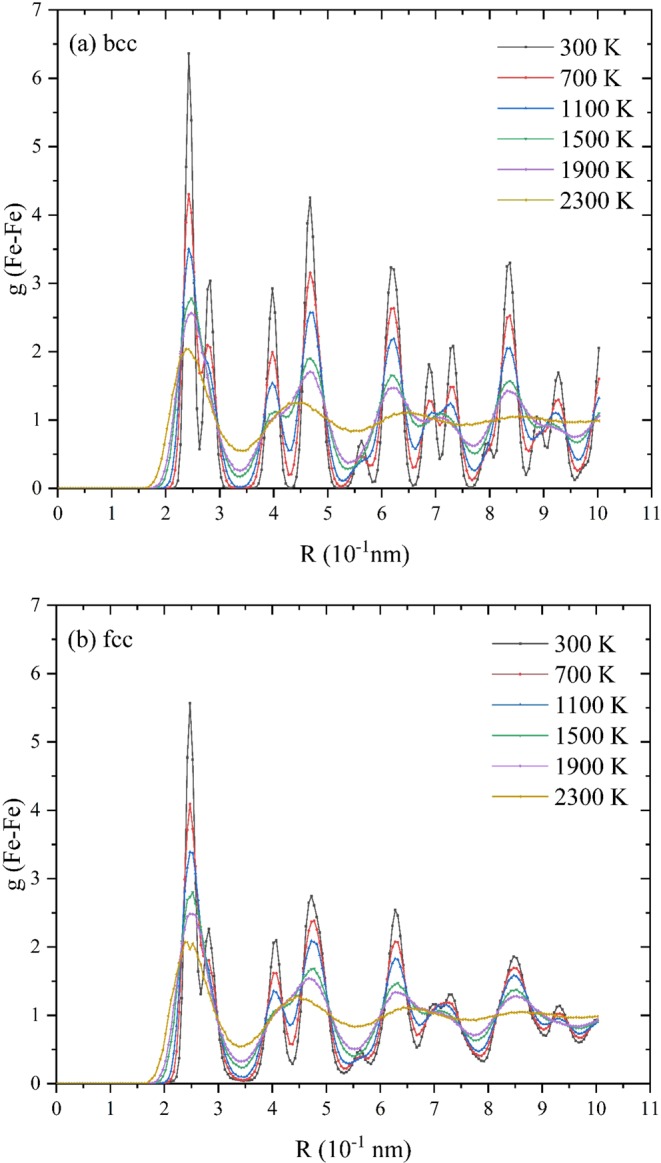
RDF results of (**a**) bcc and (**b**) fcc bulk iron during the melting tests with 400 K interval.

Radial distribution function (RDF) is introduced to study the structural evolution of both materials during the melting process^21^:

3 $${\rm{g}}(r)=\frac{1}{\rho 4\pi {r}^{2}}\frac{{\sum }_{t=1}^{T}{\sum }_{j=1}^{N}\Delta N(r\mathop{\to }\limits^{\Delta }r+dr)}{N\times T}$$

in which T represents the total computational time, N means the total number of atoms, ρ denotes the system quantity density, and r denotes the radius between the latter atom a and the reference atom b. RDF results of bulk materials in melting process are shown in Fig. 2. Under lower temperatures (300 K- 1100 K), both bcc and fcc show regulate structures which indicates they maintain lattice features within the temperature range. After the temperature exceeds 2100 K, both RDF curves of fcc and bcc become more flat: except the first peak, every two adjacent peaks combine into a single peak and the peak value of curves are almost 80% lower than that of the 300 K curve which indicate the crystal has lost its lattice characters and enters into a state of complete melting.

It has been reported that a crystal can be superheated significantly above its melting temperature. Once the nucleation of liquid initiates the complete melting occurs^22^. To obtain the real melting points calculated by MD simulations, we have to eliminate effects caused by superheating phenomenon. According to Fig. 1(a,b) Our results show the limit of superheating (T_LS_) for bcc and fcc bulk iron are 2125 K and 2150 K respectively. According to the ref. ^22^, melting temperatures obtained by MD simulations should be modified by Eq. 4:

4 $$\frac{{T}_{LS}}{Tm}-1=\frac{ln2}{3}\,$$

where T_m_ represents the melting temperature. After revising, T_m_ for bcc and fcc crystal predicted by ReaxFF force field should be 1746.55 K and 1726.24 K respectively.

In Table 4 we compare our results with other literature. In the table, SC notes the Sutton-Chen potential, and FS notes the Finnis-Sinclair potential. It is obvious that, although the Reaxff force field overestimates the melting points compared with experimental results, it still produces better results than EAM, FS and PS force field.

**Table 4:** Melting points of bulk iron calculated by different authors with different techniques.

	Potential	Structure	T_m_ (K)
Experimental^23^	—	bcc	1811
Phase diagram^24^	—	fcc	1800.8
This work	ReaxFF	bcc	1746.55
	ReaxFF	fcc	1726.24
Shen *et al*.^25^	SC	fcc	1833.3
Sun *et al*.^26^	EAM	bcc	2358.7
	EAM	fcc	2251.0
	Pair	bcc	2311.8
	Pair	fcc	2202.0
Shibuta *et al*.^27^	FS	bcc	2400.0
Shibuta and Suzuki^6^	FS	bcc	860.0

### Effect of size on Fe nanoparticles melting

Generally speaking, there are four methods to judge melting points of materials in MD: plots of potential energy, system heat capacity C_v_, Lindemann index^28^ vary with temperature and equilibrium temperature of solid-liquid interface. Considering that our research focuses on nanoparticles and their energy properties, we adopt the first three method to study melting behaviors of FNPs in different perspectives.

**Figure 3:**
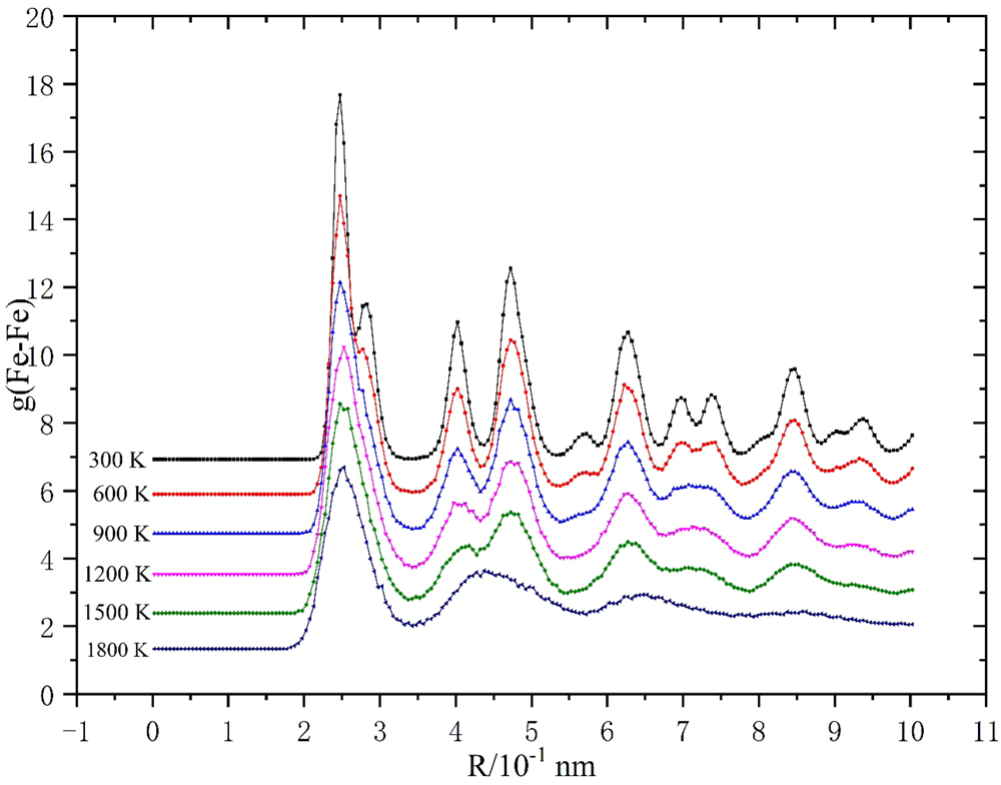
Radial distribution function curve of 2 nm FNP in heating process before 1,800 K.

The RDF results of Fe-Fe pair before and after 1,800 K are shown in Fig. 3.

It is obvious that the first peaks of all plots appear near R=2.87 Å which is the lattice parameter of bulk iron. Note that, for the perfect crystal structure, the radial distribution function is characterized by periodicity which indicates the distance between peaks represents the lattice parameter of the crystal. When the temperature is low (before 1,000 K), the peak characteristic of plots is obvious, and all the peaks are sharp compared with other parts of plots. The increase of temperature results the decrease or disappearance of peak height. The disappearance of RDF peak could be regarded as the symbol of melting taking place in this region. The radical distribution curve before melting presents similar distribution characteristics to bulk crystals: the peak is obvious and arises periodically. As the temperature rises, RDF plots become smooth slowly from the surface slowly from the surface region to the center of particle. Furthermore, the value of RDF plots decreases but maintains a sharp peak near the center of particle. Before melting (1,800 K), the value of RDF persists declination. Simultaneously, the curves extend to the region far from the center. It is worth noting that, when temperature is 300 K, there is a tiny peak adjacent to the first one which is located 0.25 Å further than that of the first peak. The first peak represents the broadest Fe -Fe pair in a perfect lattice which mainly exist in the interior region of FNPs. The tiny peak could be regarded as the surface Fe atoms which are characterized by less coordination numbers. Additionally, this tiny peak is also the first to disappear before 1,000 K. All above analyses prove the pre-melting process is generated from the surface and propagates to the interior.

Traditionally, the melting behaviors are decided by the change of potential energy curves. The melting process undergoes the evolution of solid, solid-liquid coexistence, liquid states, which can be shown from the potential energy curves. Besides potential energy studies of FNP melting, thermal property caused by melting is also considered. The system heat capacity, C_v_, which is another important parameter for most energy systems represents the variation of particle energy:

5 $${C}_{v}=\frac{{\langle {E}_{t}^{2}\rangle }_{T}-{\langle {E}_{t}\rangle }_{{T}^{2}}}{2N{k}_{B}{T}^{2}}$$

where K_B_ is the Boltzmann constant, E_t_ is the total system energy. T is the system temperature and < >_T_ notes the ensemble average value (an average of a period of equilibration). The C_v_ value is expected to increase rapidly when temperature reaches the melting point and remains stable in other temperatures.

**Figure 4:**
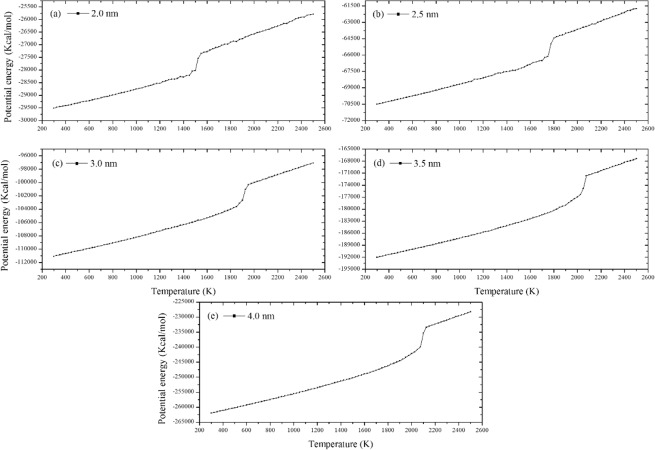
Curves of potential energy of FNPs (E_pot_) very with time (**a**–**e**): The diameter of FNP ranges from 2.0 nm to 4.0 nm.

**Figure 5:**
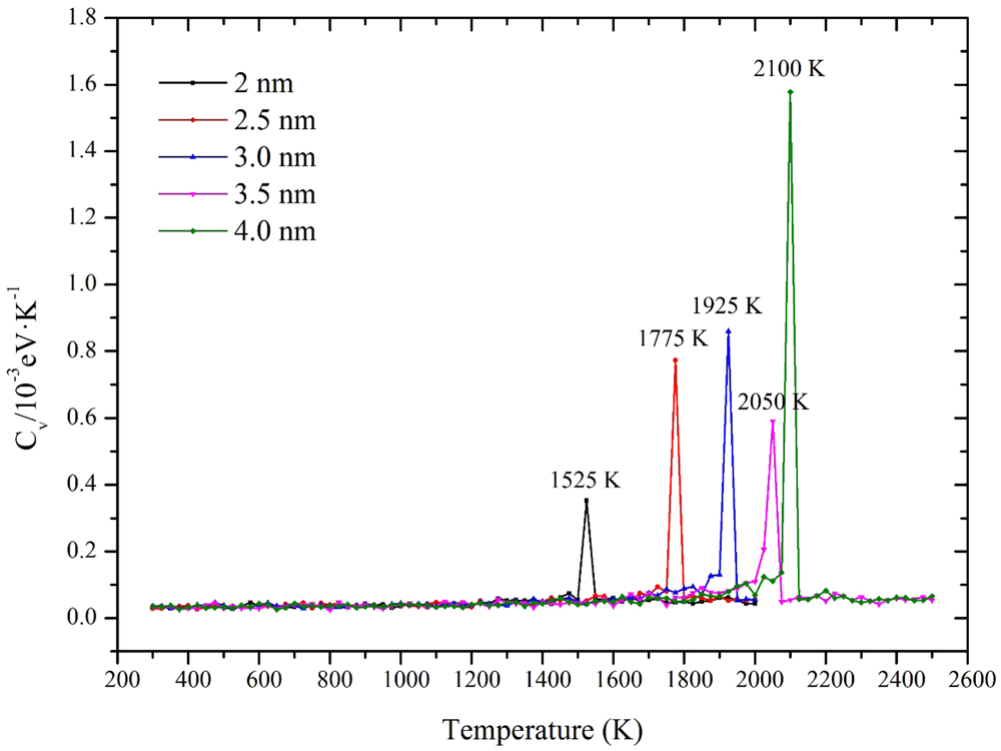
The heat capacities (Cv) of different size FNPs as a function of temperature.

The potential energy of FNPs could give us a direct indication of phase transition during the melting processes as Figs. 4 and 5 show vary with time.

From Fig. 4, the potential energy curve of 2.0 and 2.5 nm FNPs show tiny fluctuation. Such phenomenon is known by the bistable regime which means both solid and liquid are coexistent in the temperature range. However, when FNP particle diameter is larger than 2.5 nm, there are no bistabile states observed and the potential energy changes smoothly. Such findings are also corresponded with Samam *et al*.’s results calculated by ES+ potential MD simulations^29^. Figure 5 clearly shows the melting points of each FNP by the temperature of occurrence of peak values. The melting points predicted by Cv and potential energy plots are consistent. Limited by the accuracy of temperature rise 25 K, the height of peak has no scientific reference significance.

Different from the change of potential energy and C_v_, the Lindemann index tells structure changes of FNPs in melting processes.

6 $${\rm{\delta }}=\frac{2}{N(N-1)}{\sum }_{i < j}\frac{\sqrt{{\langle {r}_{ij}^{2}\rangle }_{t}-{\langle {r}_{ij}\rangle }_{t}^{2}}}{{\langle {r}_{ij}\rangle }_{t}}$$

where r_ij_ is the distance between atom i and j. <>_t_ denotes a time average at the current temperature. The value of Lindemann index is expected to increase abruptly by a factor when the system is heated to the solid-liquid coexistence state and the temperature will be regarded as the melting point of the nano-particle^30^.

**Figure 6:**
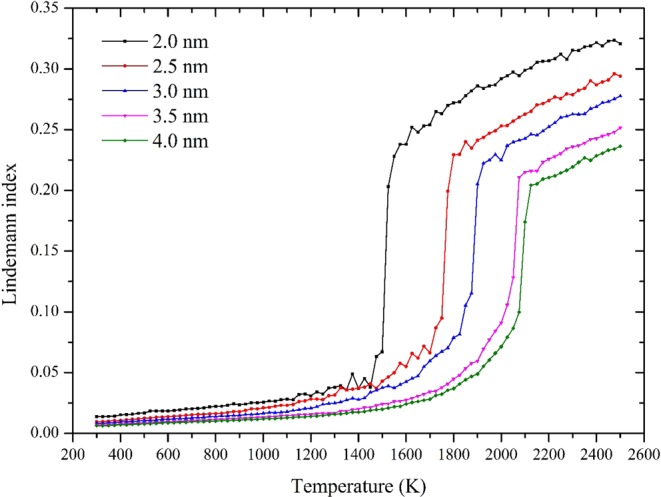
Lindemann index of different size FNP as a function of temperature (T).

Figure 6 shows calculation results of Lindemann index. Similar to the potential energy plots, for 2.0 nm and 2.5 nm, tiny fluctuations of are detected before melting and Lindemann index varies with temperature relatively smoothly for larger particles.

It is worth noting that we still lack data from experiments to decide FNP melting points. Due to the fact that nanoparticles could have numerous isomers and the whole system could be trapped in any one of these possible configurations. Therefore, one will obtain melting points of nanoparticles by averaging all melting points of those isomers which is complicated in experiments. Based on the above analysis ReaxFF successfully detect the size effect and structure evolution of FNPs in melting process. Additionally, we also confirm melting rule of FNPs is not a simple linear relationship which is also consistent with findings in replica exchange molecular dynamics simulations^5^.

### Fe nanoparticles melting with different defect concentrations

After validating the reliability of the ReaxFF force field for Al melting process, we study melting behaviors and energy characteristics of FNPs with different defect concentrations. In industry production, electrical explosion is one of the main methods to product FNPs in a large scale^31,32^. FNPs produced by this method are reported to be characterized by different defect concentrations. Effect of voids on melting has ever been studied by Puri *et al*. However, they modeled metal clusters with defects by cutting regular cubes containing certain number of atoms^33^. Such modeling approach may not reflect the actual situation scientifically. To clarify how defects affect the energy of FNPs in melting process, we introduce the concept of extra storage energy E_e_ which could be expressed as:

$${E}_{e}=\frac{{E}_{tot}-N\times \varepsilon }{N}\times {N}_{A}$$

7 $${\rm{\varepsilon }}=\frac{{E}_{t}}{N}$$

where N_A_ is the Avogadro constant and ε represents the binding energy of bulk iron without defects at current temperature.

**Figure 7:**
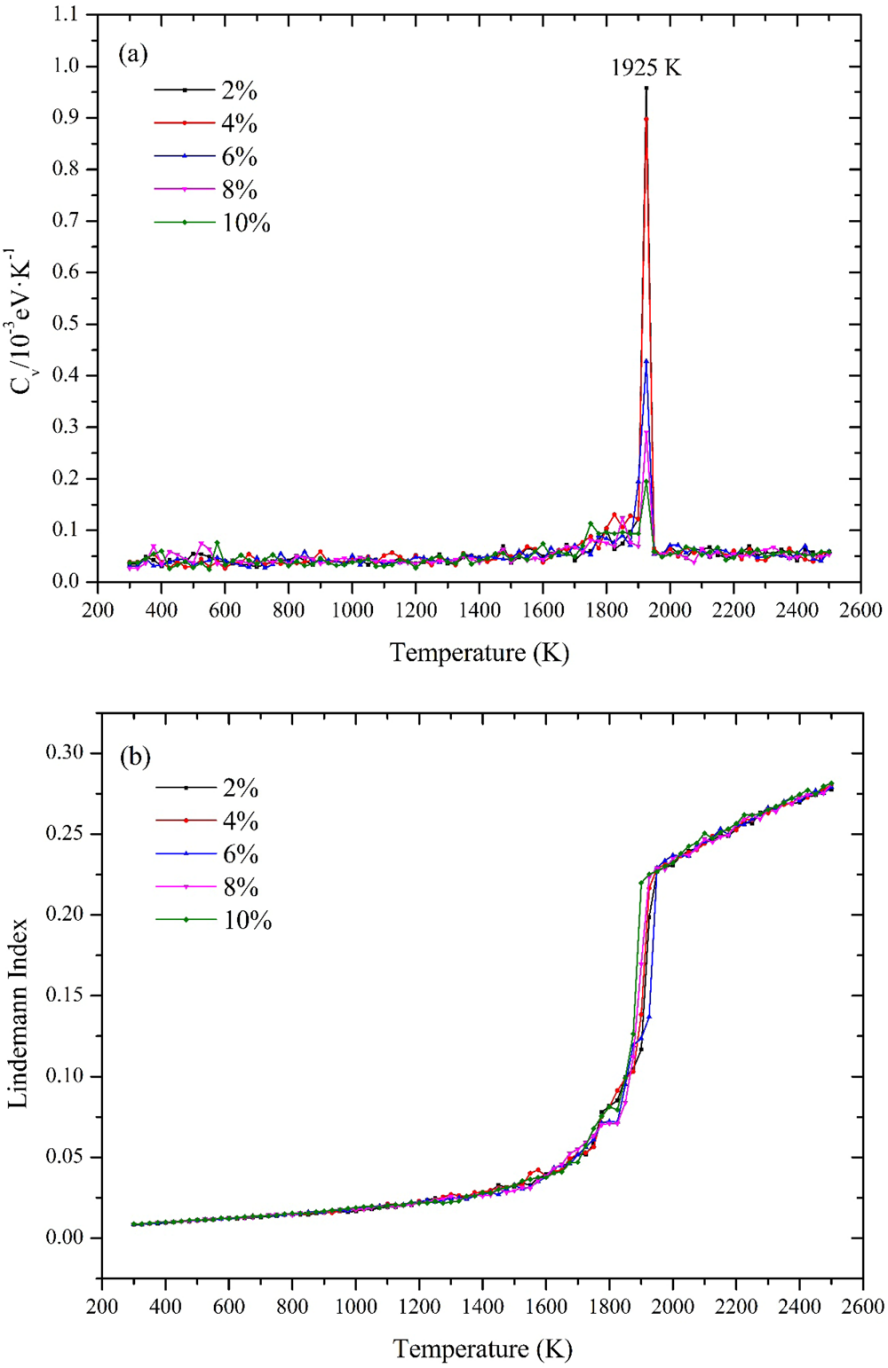
(**a**) The heat capacities (C_v_) of FNPs with range form 2% to 10% as a function of temperature. (**b**) Plots of Lindemann index varies with temperature.

Firstly, we predict the melting points of FNPs with different defect concentrations. Heat capacity and Lindemann index are adopted to respectively in Fig. 7(a,b) respectively. All plots reach its peak value at temperature 1925 K which is the same as that of 3 nm. Similar conclusion could also be drawn from Fig. 7(b). The Lindemann index tell us the change of solid-liquid structure state. Such phenomenon proves that up to 10% of defects will not affect the melting point of FNPs. However, the total energy of them has changed a lot.

**Figure 8:**
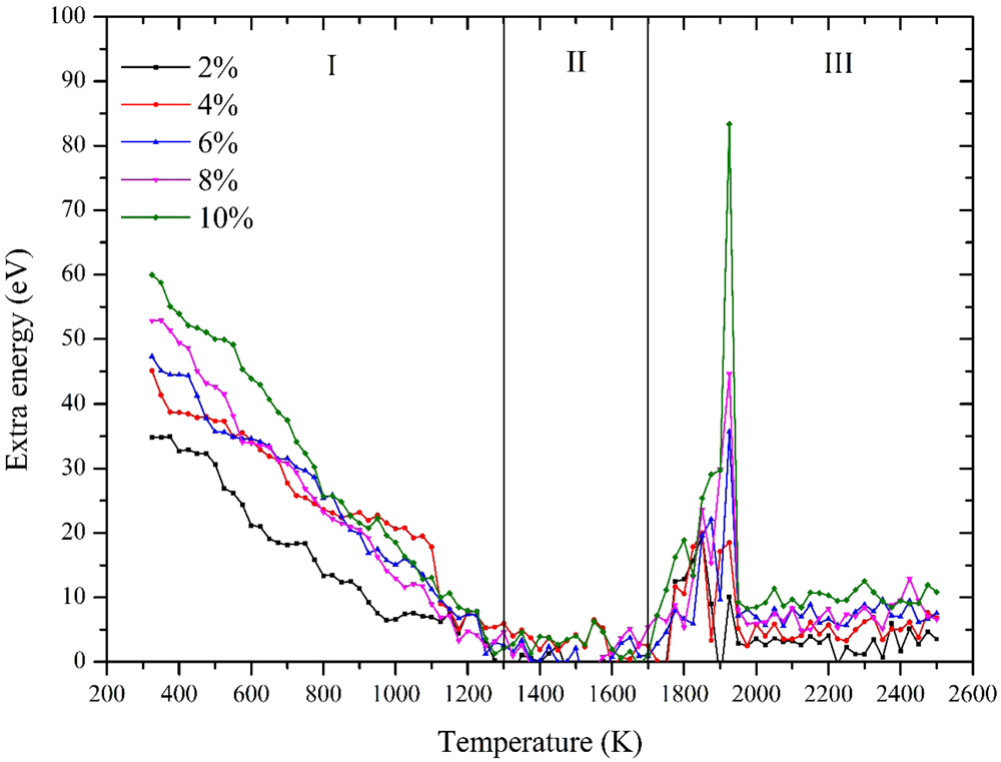
Extra energy storage plots of FNPs with different defect concentrations.

Extra energy storage of FNPs with different defects is studied according to the Eq. 6. Note that, all FNP models with defects were built based on the concept that the diameter of particle is retained at 3 nm but only a certain number of interior atoms was deleted randomly inside. As a result, we can refer to the 3 nm FNP to study thermodynamic properties of FNPs with defect concentrations. Figure 8 shows how extra energy storage of FNPs with defects change during the melting process.

According to the changing trend of plots, we divide extra storage energy plots into three stages. Firstly, at room temperature 300 K, the extra storage energy increases with the increase of defects. On average, every 2% defect concentration products 5 eV of extra energy. Before 1300 K, extra energy for all cases decrease with increase of temperature and the average decline rate ranges from 0.0354 eV/K to 0.0578 eV/k. After that all cases enter the second stage in which temperature ranges from 1300 K to 1700 K. In this stage, all extra energy plots fluctuate around a certain value keep at a relative low level: less than 10 eV. When the temperature approaches the melting point, extra energy begins to rise at a relative high rate and achieve its peak value at melting point 1925 K. Additional energy storage produces peak at melting points is beneficial to fuel because nanoparticles are well known that they will be ignited at their melting points. After melting, all extra energy plots maintain a relative high level with fluctuations. Detail information in three stages mentioned above is listed in Table 5. Such extra storage energy characteristic of FNPs with defects will explain why FNPs have better ignition and combustion performance as an additive of solid propellants.

**Table 5:** Key properties of FNPs with defects in three stages.

Defect concentration	2%	4%	6%	8%	10%
I. Decline rate (10^−2^ eV/K)	3.54	3.91	4.47	4.83	5.78
II. Average extra energy (eV)	−1.59	0.14	0.71	0.74	2.8
III. Peak value (eV)	10.12	18.51	35.71	44.61	83.35

## Conclusions

In this paper, we perform ReaxFF MD simulations to study melting behaviors of FNPs with/without defects. The force field has been tested to be valid on the bulk material firstly. ReaxFF force field can also detect the structure evolution of fcc crystal during the heating process. Pre-melting of FNPs has been proved by RDF analysis which indicates melting processes of FNPs propagate from the surface to the interior. The ReaxFF force field successfully detect the size effect of FNPs melting and prove the melting law is not linear. Defect of FNPs (0%-10%) will not affect melting points compared with that of FNPs with the same particle size. All FNPs with defects show extra storage energy in various degree. The whole process is chartered as three stages: In the first period, the extra storage energy decreases uniformly with the increase of temperature and almost disappear near 1300 K; After that, extra storage energy maintains relative low level until melting; The peak value appears at the melting point for all FNPs with defects After melting, all samples completely become liquid but always remain positive extra energy accompanied by fluctuations. Our research could be instructive for the future application of using ReaxFF force field to study the combustion of FNPs as a novel solid propellant fuel.
